# *Echinophyllia tarae* sp. n. (Cnidaria, Anthozoa, Scleractinia), a new reef coral species from the Gambier Islands, French Polynesia

**DOI:** 10.3897/zookeys.318.5351

**Published:** 2013-07-24

**Authors:** Francesca Benzoni

**Affiliations:** 1 Department of Biotechnology and Biosciences, University of Milano-Bicocca, Piazza della Scienza 2, 20126 Milan, Italy

**Keywords:** Lobophylliidae, *Echinophyllia echinata*, *Echinomorpha nishihirai*, Tara Oceans Expedition

## Abstract

A new shallow water scleractinian coral species, *Echinophyllia tarae* **sp. n.**, is described from the Gambier Islands, French Polynesia. It is characterized by an encrusting corallum, a few large and highly variable corallites with protruding walls, and distinctive costosepta. This coral was observed in muddy environments where several colonies showed partial mortality and re-growth. The new species has morphological affinities with both *Echinophyllia echinata* and with *Echinomorpha nishihirai*, from which it can be distinguished on the basis of the diameter and the protrusion of the largest corallite, the thickness of the septa, and the development of the size of the crown of paliform lobes.

## Introduction

At present, *Echinophyllia* Klunzinger, 1879 is known to include eight extant zooxanthellate species (Veron and Pichon 1980, Veron 2000, 2002), namely *Echinophyllia aspera* (Ellis & Solander, 1786), the type species, *Echinophyllia echinata* (Saville-Kent, 1871), *Echinophyllia orpheensis* Veron & Pichon, 1980, *Echinophyllia echinoporoides* Veron & Pichon, 1980, *Echinophyllia patula* (Hodgson & Ross, 1982), *Echinophyllia costata* Fenner & Veron, 2000, *Echinophyllia pectinata* Veron, 2000, and *Echinophyllia taylorae* (Veron, 2000). The genus used to be part of the family Pectiniidae Vaughan & Wells, 1943 together with *Pectinia* Blainville, 1825, *Mycedium* Milne Edwards & Haime, 1851, and *Oxypora* Saville-Kent, 1871. Based on molecular results by Fukami et al. (2008), Dai and Horng (2009) moved *Echinophyllia* and *Oxypora* to the family Lobophylliidae Dai & Horng 2009, and Budd et al. (2012) placed the remainder of the Pectiniidae in the Merulinidae Verrill, 1866. To date, the most detailed and updated overview of morphologic characters of *Echinophyllia* including micro-morphology is provided by Budd et al. (2012). However, species level morpho-molecular investigations of species boundaries and phylogenetic relationships for *Echinophyllia* have not been performed, and so far the genus has never been formally revised.

Presently, *Echinophyllia* is known to occur in the Indo-Pacific, from the seas around Arabia (Sheppard and Sheppard 1991, Pichon et al. 2010, Riegl et al. 2012), the Indian Ocean (Obura 2012) and the western and central Pacific Ocean (Chevalier 1971, Veron and Pichon 1980, Best et al. 1989, Veron 2000, Hoeksema and van Ofwegen 2004) to French Polynesia in the east, including the Society, Tuamotu, Austral and Gambier Islands (Pichon 1985, Glynn et al. 2007).

**Figure 1.**
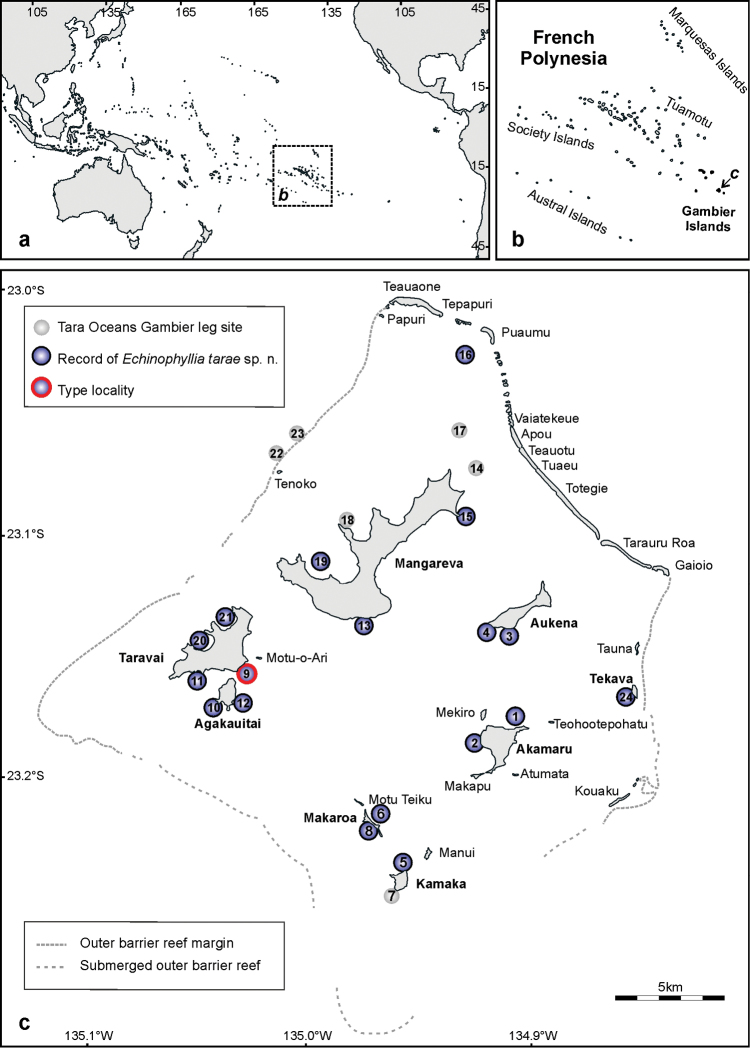
Map of **a** the Pacific Ocean, showing the position of French Polynesia **b** the island groups belonging to French Polynesia, and **c** the study area in the Gambier Archipelago showing the 24 sampling sites of the Tara Oceans leg and the sites where *Echinophyllia tarae* sp. n. was found. Stippled rectangle in **a** shows the position of the map shown in **b.** Arrow in **b** shows the position of the Gambier Archipelago shown in **c**.

The remote and relatively poorly studied Gambier Islands are found at the southeast end of the vast French Polynesian territory (Figure 1a, b). The actual islands were once all part of the same volcano, which in time has almost completely drowned (Brousse et al. 1974). Today, they are found in a lagoon approximately 35 km long (north to south) and 30 km wide (west to east) delimited by a continuous reef, which emerges at low tide in the north and is submerged in the south (Brousse et al. 1974, Chevalier et al. 1974) (Figure 1c). The current knowledge of reef-dwelling corals from the Gambier Archipelago is based on the studies carried out in the mid-seventies by Chevalier (1974), who published a preliminary list including 60 species of zooxanthellate and azooxanthellate scleractinians. Since then, no further studies were carried out on the coral fauna of the islands until the Tara Oceans scientific Expedition with MV Tara (Karsenti et al. 2011) allowed sampling of 24 sites between June and July 2011, which also resulted in an update on the local mushroom coral fauna (Hoeksema and Benzoni 2013).

With regard to *Echinophyllia* species, Chevalier (1974) only reported on the presence of *Echinophyllia aspera*. According to him this species is typical of fringing reefs around the main islands in the large lagoon. During the recent Tara Oceans Expedition the presence of *Echinophyllia aspera* was actually not recorded. However, another *Echinophyllia* species, morphologically different from others already known, was commonly observed at the fringing reefs and lagoon pinnacles of the Gambier Islands. The species is here described as *Echinophyllia tarae* sp. n. and its similarities with its congeners as well as *Echinomorpha nishihirai* (Veron, 1990) are discussed.

## Methods

A reference collection of Scleractinia was sampled in the Gambier, including coral skeletons and tissues fixed for DNA, after *in situ* photographs were taken. The collection contained five specimens of *Echinophyllia tarae* sp. n. Sampling took place during SCUBA diving at different sites around the islands of Taravai, Akamaru, and Makaroa (Figure 1). Digital images of living corals in the field were taken with a Canon G9 in an Ikelite underwater housing system. Coral specimens were collected, tagged, and for each specimen a fragment of 1 cm^2^ was broken off the colony and preserved in CHAOS solution for further molecular analysis. The remaining corallum was placed for 48 hours in sodium hypochlorite to remove all soft parts, rinsed in freshwater and dried for microscopic studies. Images of coral skeletons were taken with a Canon G9 digital camera and through a Leica M80 microscope equipped with a Leica IC80HD camera. For high resolution and deep field close ups of three-dimensional details of corallites and septa, a series on images of the same subject at different focus intervals were taken (approximately 10) and the images were fused using the Helicon Focus 5.3 software (Kozub et al. 2000–2012).

A total of 24 sites was surveyed (Table 2). Each site position was recorded with a Garmin eTrex GPS. At each site all coral species encountered in a 1 hour SCUBA dive were recorded and data were included into a geo-referenced database. At least two images per specimen were taken underwater, one of the complete colony and one close-up. Digital images were then analyzed to verify underwater preliminary records and species presence records were used to produce a species per site matrix. Data on the occurrence of *Echinophyllia tarae* sp. n. in the field in the different sites was extracted from this species per site database.

The holotype was deposited at Museum National d’Histoire Naturelle (MNHN) in Paris, the other four specimens are at the University of Milano-Bicocca (UNIMIB) coral facility together with the rest of the Tara Oceans Expedition collection (186 specimens), which will ultimately be housed at the MNHN once their study is completed. Specimens of other *Echinophyllia* species were examined at the Museum of Tropical Queensland (MTQ), Townsville, Australia, and at the Institut de Recherche pour le Développement (IRD), Nouméa, New Caledonia.

## Systematic section

### Order Scleractinia Bourne, 1905
Family Lobophylliidae Dai and Horng, 2009
Genus *Echinophyllia* Klunzinger, 1879

**Type species** (by monotypy). *Madrepora aspera* Ellis & Solander, 1786.

#### Echinophyllia tarae sp. n.

urn:lsid:zoobank.org:act:9C41F53B-3FEE-47BA-8017-48C252F65F5E

http://species-id.net/wiki/Echinophyllia_tarae

Figures 2 –8 , 9a, b , 10b, d

##### Material examined.

**Figure 2.**
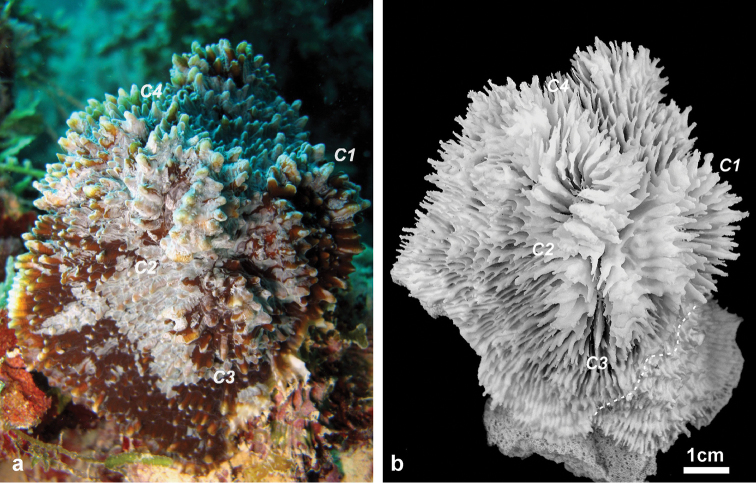
Holotype of *Echinophyllia tarae* sp. n. (MNHN-IK.2012–8000) **a** the colony *in situ* before collection, and **b** the corallum after removal of the animal tissues. C1 to 4 indicate the position of same corallite (C) in the two images. Numbers are assigned in decreasing order of corallite size, C1 being the largest. Stippled line on the specimen in **b** shows the boundary of living tissue at the time of collection.

**Figure 3.**
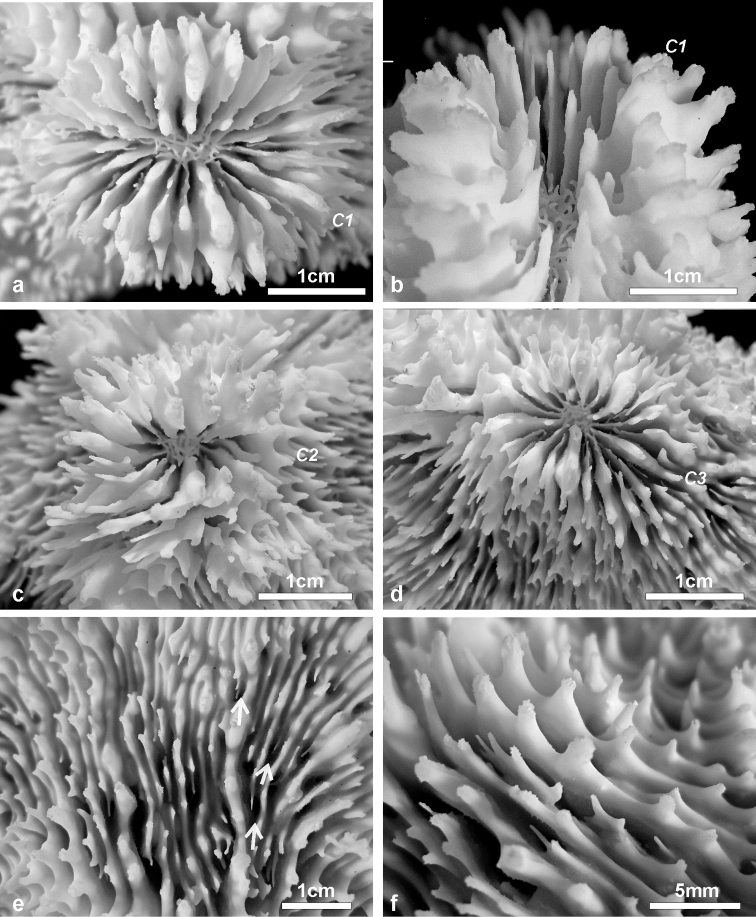
Details of corallites, septa, and costae in the holotype of *Echinophyllia tarae* sp. n. (MNHN-IK.2012–8000) **a** top view of the largest corallite in the colony, **b** lateral view of the same corallite shown in **a**, **c** top view of the second largest corallite, and **d** of the third **e** top and **f** side view of the costae. C1 to 3 indicate the corallites as shown in Figure 2. White arrows in **e** indicate the position of exothecal alveoli at the insertion of costae.

**Figure 4.**
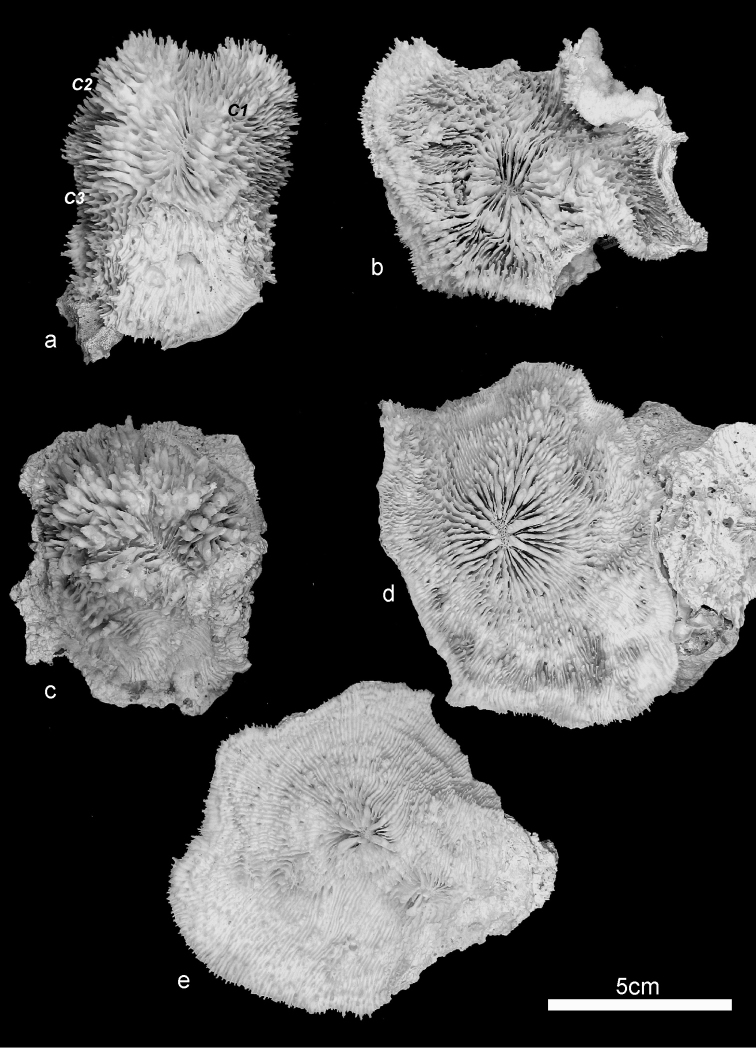
Specimens of *Echinophyllia tarae* sp. n. collected for this study **a** side view of the holotype (MNHN-IK.2012–8000) **b** specimen UNIMIB TO-GA028 **c** specimen UNIMIB TO-GA186 **d** specimen UNIMIB TO-GA099 **e** specimen UNIMIB TO-GA071. C1 to 3 indicate the holotype corallites as shown in Figures 1 and 2.

**Holotype:** MNHN-IK.2012-8000 (Figures 2–4). Type Locality: Taravai Island, Gambier, French Polynesia (MV Tara, Tara Oceans Expedition, Site 9), 23°9.404'N, 135°1.769'E, 10 m, 30 June 2011, coll. F. Benzoni.

*Corallum*: The holotype is a knob-shaped, plocoid, encrusting colony attached to a fragment of a dead tabular *Acropora* coral (Figure 2). The specimen is 9.2 cm high, and 8.5 x 5 cm wide at the base in its original growth position.

*Corallites*: The 12 corallites are oval in shape and variable in size (Figure 2, 3), ranging from 3.3 cm in diameter (C1 in Figure 2, 3a) to 1.0 cm. Corallites are organically united (see terminology in Budd et al. 2012). The central position of C1 (the largest corallite) is less obvious in the holotypes due to its knob-shaped growth form. Corallites protrude up to 1 cm and are directed in different directions (Figure 2). Corallite wall is septothecal.

*Costosepta*: Variable in number depending on the size of the corallite (Figure 3a–d), exsert and thickened over the theca. The largest corallite contains 26 septa arranged in five orders (Figure 3a). Septa of the first three orders are thicker than the others. Septal teeth are elliptical in outline, large and high (> 0.6 mm) according to the parameters defined by Budd et al. (2012) (Figure 3a–d), and their tips are irregular bulbous. Tooth spacing is very high (> 2 mm). Septal side granulation is weak. Paliform lobes (see Benzoni et al. 2011 for a definition) thick and well developed, forming an obvious crown around the columella, which was also visible *in vivo*. Paliform lobes always present and of similar size at the proximal margin of the first two orders of septa (Figures 3a–d). In larger corallites, in which more than four orders are present, they can also form in front of the third order but then they are of smaller dimensions (Figure 3b). The costal parts of the costosepta are thick and unequal. They are strongly ornamented by triangular shaped teeth (Figure 3e, f), which bear fine granules on the tip (Figure 3f). Exothecal alveoli are present at the insertion of lower order costae (Figure 3e). Costae cover the whole surface of the coenosteum between corallites.

*Columella*: Well developed, deep in the fossa (Figure 3b) made by a mesh of twisted intermingled processes derived from the inner end of the higher order septa: the first two in smaller corallites (Figure 3a–d), and up to the fourth order in larger corallites (Figure 10b–d).

*Colour*: The living colony was mottled brown. Tips of septa and costae ornamentation varied from light beige to white.

##### Other material

**(Gambier, French Polynesia, Tara Oceans Expedition):** UNIMIB TO-GA028, Akamaru Island (Site 2), 23°11.082'N, 134°54.331'E, 26 June 2011, coll. F. Benzoni; UNIMIB TO-GA071 Makaroa Island (Site 6), 23°12.960'N, 134°57.991'E, 28 June 2011, coll. F. Benzoni; UNIMIB TO-GA099 Taravai Island (Site 11), 23°9.540'N, 135°3.055'E, 1 July 2011, coll. F. Benzoni; UNIMIB TO-GA186 Taravai Island (Site 9), 23°9.404'N, 135°1.769'E, 30 June 2011, coll F. Benzoni.

**Figure 5.**
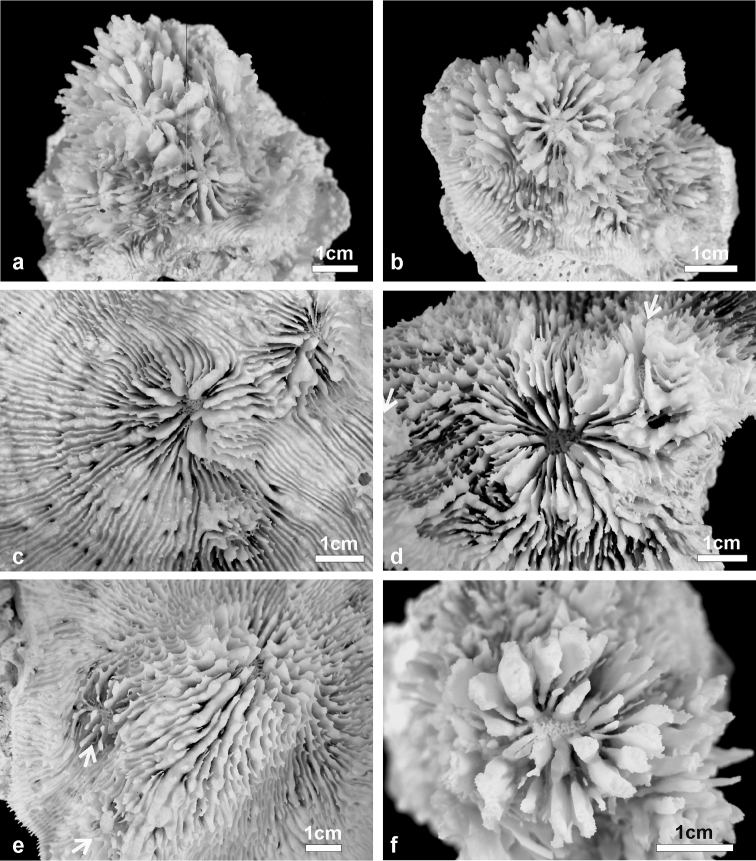
Variation in shape and size of the corallites of *Echinophyllia tarae* sp. n. **a** and **b** two lateral views of specimen UNIMIB TO-GA186 **c** specimen UNIMIB TO-GA071 **d** UNIMIB TO-GA028 **e** specimen UNIMIB TO-GA099 **f** close up-of a corallite of the same specimen as in **a** and **b**. White arrows in **e** indicate the position of secondary corallites.

**Figure 6.**
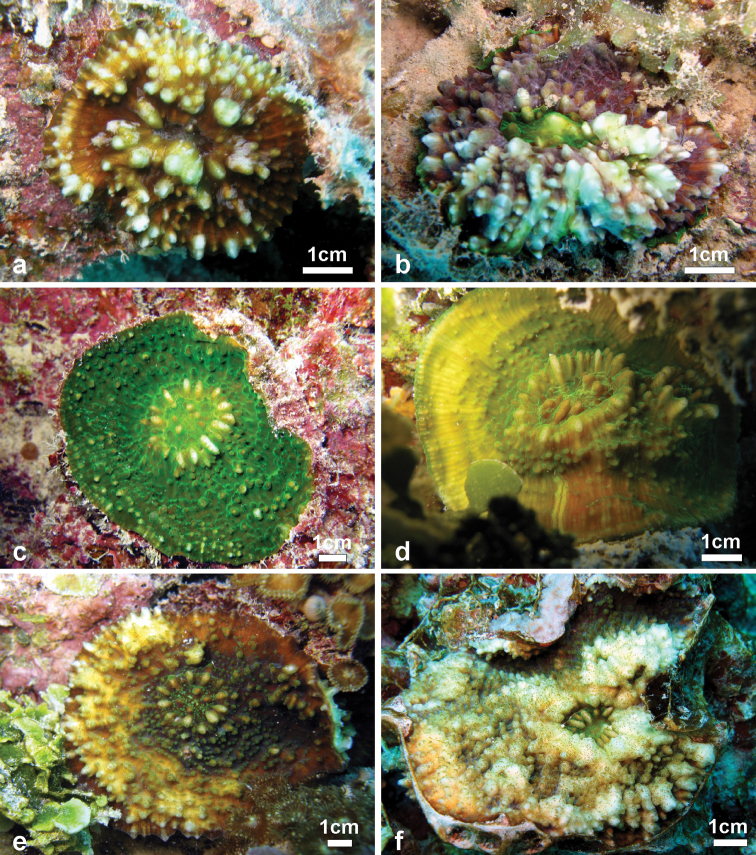
Variation of corallum shape, costosepta ornamentation, and colouration in smaller colonies of *Echinophyllia tarae* sp. n. observed *in situ* **a** young one polyp brown coloured specimen settled on crustose coralline algae, Mangareva Island (Site 15) **b** a dark brown two polyp colony with green oral discs, Taravai Island (Site 21) **c** one polyp bright green specimen with a large raised central corallite in which the crown of pali is clearly visible and spiky and well developed costae, Makaroa Island (Site 6) **d** a light green colony with two raised polyps in central position, well developed crown of pali and costae ornamentation smoothening towards the colony periphery, northern lagoon pinnacles (Site 16) **e** brown colony with one raised polyps in central position and markedly teethed costae, Mangareva Island (Site 13) **f** *in situ* image of specimen UNIMIB TO-GA028 (Figures 4b, 5d) showing the irregularly shaped colony with a very rugged and almost inflated appearance, Akamaru Island (Site 2). Sites are indicated in Figure 1.

**Figure 7.**
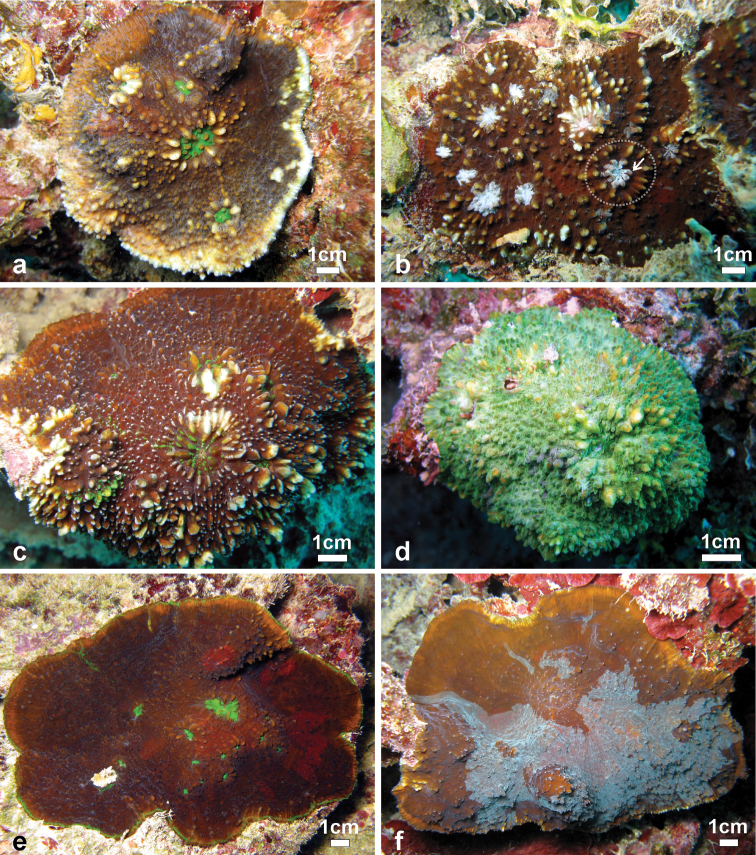
Variation of shape, spikiness of septa and costae, and colouration oflarge colonies *Echinophyllia tarae* sp. n. observed *in situ* **a** brown encrusting colony with free margins, bright green oral discs and raised corallites, Akamaru Island (Site 2) **b** brown encrusting colony with white oral discs, raised corallites (larger one in the stippled circle), and very spiky costae, Taravai Island (Site 9 – type locality), the prominent crown of paliform lobes of the largest corallite is indicated by the white arrow **c** brown knob shaped colony with bright green oral discs and raised corallites, note the white colouration of the tips of costae teeth, Taravai Island (Site 9 – type locality) **d** a bright green knob shaped colony, Taravai Island (Site 9 – type locality) **e** brown encrusting colony with bright green oral discs and relatively low-lying corallites, note the uniform colouration of the costae, Taravai Island (Site 9 – type locality) **f** mottled brown encrusting colony with free margins and relatively low-lying corallites, note the uniform colouration of the costae, Taravai Island (Site 9 – type locality). Sites are indicated in Figure 1.

**Figure 8.**
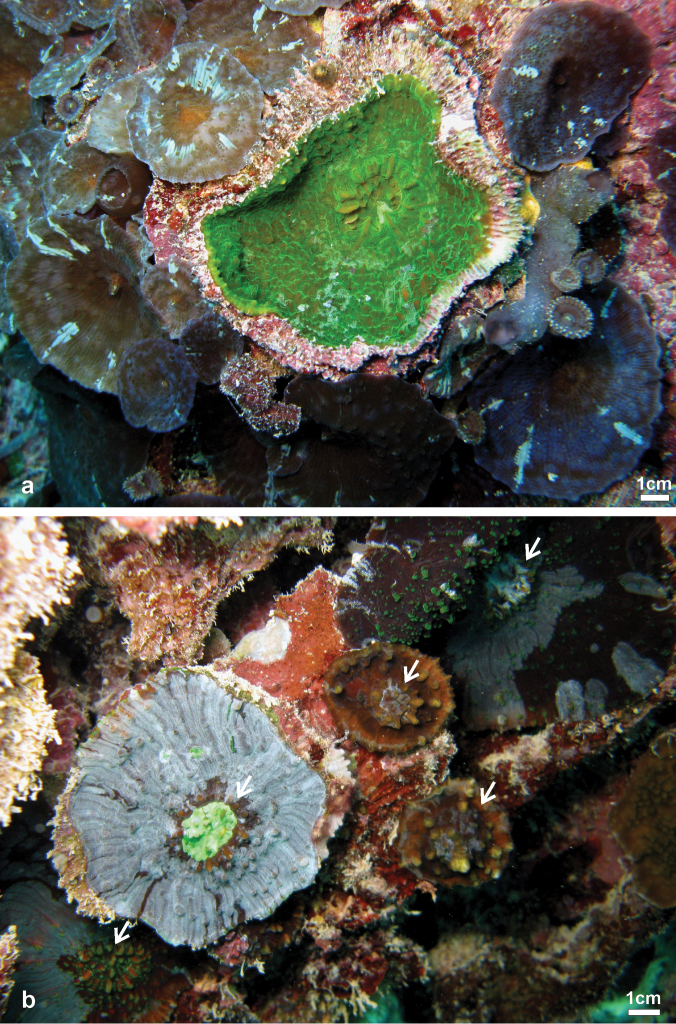
Frequently observed patterns of partial death and re-growth of *Echinophyllia tarae* sp. n. in the field**a** concave colony with a large central corallite showing a peripheral rim of skeleton encrusted by pink coralline algae and surrounded by zoanthids and corallimorpharians, Mangareva Island (Site 19) **b** a similar situation as in **a** but with re-growth occurring over previously dead colonies, note the variation of colouration in adjacent corals, Taravai Island (Site 11). White arrows in **b** indicate the position of larger central corallites. Sites are indicated in Figure 1.

*Variation of skeletal structures*: Colony size is relatively small (Figures 2–8), the largest colony is 20 cm wide (Figure 6f). Corallum generally encrusting, its plane following the surface of the underlying substrate (Figures 4b, d, e) but also knob-like (Figures 4a, c) with foliose margins where they become detached from the substrate. The number of corallites per colony is low, ranging from 1 to 15. A large, central primary corallite is always present (Figures 2, 6a, b). Secondary corallites are often inclined in various directions and show variable diameter sizes within the same colony (Figures 5a–e, Table 1). The largest corallite observed (specimen UNIMIB TO-GA099, Figure 4d) is 3.5 cm in diameter, the smallest, in the same specimen, 0.9 mm (Figure 5e). The numbers of septa, orders of septa, and paliform lobes vary between and within colonies. First order septa always thicker than the others and in some cases up to 4 mm thick (Figure 5f). Columella always present, large and oval in the largest corallite, less developed is smaller corallites. Costae typically thick, alternating in size (Figure 5c, d) and strongly dentate, although variably so between specimens.

**Table 1.** Dimensions of the examined specimens of *Echinophyllia tarae* sp. n. For each specimen the total number (n.) of corallites, the maximum and minimum diameter of the largest and smallest corallite in the colony, the number of septa, and of paliform lobes is listed. LC = largest corallite; SC smallest corallite.

**Specimen code**	**n. of corallites**	**Diam. LC max-min: (cm)**	**Diam. SC max-min: (cm)**	**n. of septa LC**	**n. of paliform lobes LC**
MNHN-IK.2012–8000	12	3.3 – 2.0	1.0 – 0.9	26	11
UNIMIB TO-GA028	4	3.1 – 2.4	1.1 – 0.9	39	14
UNIMIB TO-GA071	4	2.3 – 2.1	1.0 – 0.9	34	8
UNIMIB TO-GA084	4	3.2 – 2.4	1.9 – 1.8	29	11
UNIMIB TO-GA099	3	3.5 – 3.0	1.2 – 1.0	52	24

*Field characteristics and colouration*: The colouration is showing much variation, ranging from dark brown (Figures 7a–c, e), to mottled brown (Figures 2a, 7f), beige (Figures 6e, f), and bright green (Figures 6c, 7d). The tissue on the tips of septal teeth and costal spines teeth can be lighter in colour (Figure 6c) or white (Figure 7c), possibly as a result of tissue being less thick above these structures. In very mottled colonies (Figure 7f) or with lighter colouration of the tissue over costoseptal teeth (Figure 7b) the size and shape of the corallites may be hard to detect. The crown of paliform lobes is always prominent (Figure 5) and often obvious, especially in the largest corallite (Figures 6, 7b).

*Ecology*: *Echinophyllia tarae* sp. n. inhabits protected reef habitats and was observed between 5 and 20 m depth. It commonly grows on dead coral fragments, usually parts of branching or tabular *Acropora* colonies, which are covered by crustose coralline algae and fleshy macroalgae (Figures 6–7). This species can grow on well-illuminated surfaces but also encrusts shaded underhangs. In well-lit conditions the appearance is typically corrugated (Figures 6a–b, e–f, 7a–d). However, in some cases a certain degree of inflation of the soft tissues was observed (Figures 6a–b, f), although this generally depends on the very developed ornamentation of the underlying costosepta, which is most obvious when a live colony (Figure 6f) is compared with the clean skeleton (Figures 4b, 5d). In poorly lit conditions the overall appearance is smoother and the colouration more uniform (e.g. Figures 6d, 7e–f) although the oral discs remain generally brightly coloured and different from the rest of the tissues. Re-growth of partially dead colonies, especially at the margins, is common (Figure 8). Such patters of partial death and recovery could result from competition with other benthic invertebrates, like soft-bodied corallimorpharians and zoanthids which can co-occurr with this species (Figure 8a). The observed patterns of partial death may also be caused by deposition of sediment on the living corals. In fact, *Echinophyllia tarae* sp. n. is most commonly found at sheltered sites characterized by calm water conditions and muddy sediment which could be stirred up and deposit on benthic organisms suffocating them (Erftemeijer et al. 2012). The dead parts of the corallum are generally encrusted by coralline algae over which the coral can re-grow or re-settle (Figure 8b).

*Occurrence*:This species was commonly encountered on the fringing reefs off Mangareva, Aukena, Tekava, Akamaru, Kamaka, Makaroa, Agakauitai, and Taravai islands as well as at the base of the lagoon pinnacles found in the lagoon north of Mangareva Island (Figure 1, Table 2). Its distribution outside the Gambier archipelago is unknown although it could also occur in the Austral Islands (see Discussion section). No record is known from other localities.

*Affinities*:In its encrusting growth form, and in the presence of a central larger and protruding corallite this species is similar to *Echinophyllia echinata* and *Echinomorpha nishihirai* (see Discussion). Ongoing molecular analyses will reveal the phylogenetic relationships of this species with its congeners.

**Table 2.** Occurrence of *Echinophyllia tarae* sp. n. encountered during the Tara Oceans Expedition in the Gambier Islands. For each site the site code (as in Figure 1), island name and reef type, coordinates, sampling date, and recorded presence or absence of the species are listed.

**Site**	**Island, reef type**	**Latitude, Longitude**	**Date**	***Echinophyllia tarae* sp. n.**
**1**	Akamaru, fringing reef	23°10.61'S, 134°54.37'W	26/06/11	recorded
**2**	Akamaru, fringing reef	23°11.08'S, 134°55.33'W	26/06/11	recorded
**3**	Aukena, fringing reef	23°08.44'S, 134°55.18'W	27/06/11	recorded
**4**	Aukena, fringing reef	23°08.56'S, 134°54.74'W	27/06/11	recorded
**5**	Kamaka, fringing reef	23°14.19'S, 134°57.47'W	28/06/11	recorded
**6**	Makaroa, fringing reef	23°12.96'S, 134°57.99'W	28/06/11	recorded
**7**	Kamaka, fringing reef	23°14.98'S, 134°57.80'W	29/06/11	not recorded
**8**	Makaroa, fringing reef	23°13.32'S, 134°58.34'W	29/06/11	recorded
**9**	Taravai, fringing reef	23°09.40'S, 135°01.77'W	30/06/11	recorded
**10**	Agakauitai, fringing reef	23°10.17'S, 135°02.52'W	30/06/11	recorded
**11**	Taravai, fringing reef	23°09.54'S, 135°03.05'W	01/07/11	recorded
**12**	Agakauitai, fringing reef	23°10.35'S, 135°01.99'W	01/07/11	recorded
**13**	Mangareva, fringing reef	23°08.25'S, 134°57.11'W	02/07/11	recorded
**14**	Mangareva/Totegie	23°04.78'S, 134°54.99'W	04/07/11	not recorded
**15**	Mangareva, fringing reef	23°05.45'S, 134°55.69'W	04/07/11	recorded
**16**	Lagoon pinnacles	23°01.55'S, 134°55.69'W	05/07/11	recorded
**17**	Lagoon pinnacles	23°04.12'S, 134°55.83'W	05/07/11	not recorded
**18**	Mangareva, fringing reef	23°05.57'S, 134°59.16'W	07/07/11	not recorded
**19**	Mangareva, fringing reef	23°06.14'S, 134°59.23'W	07/07/11	recorded
**20**	Taravai, fringing reef	23°08.72'S, 135°03.09'W	08/07/11	recorded
**21**	Taravai, fringing reef	23°08.03'S, 135°02.14'W	08/07/11	recorded
**22**	outer barrier north	23°04.21'S, 135°00.85'W	09/07/11	not recorded
**23**	outer barrier north	23°03.79'S, 135°00.49'W	09/07/11	not recorded
**24**	Tekava, fringing reef	23°10.13'S, 134°51.51'W	10/07/11	recorded

##### Etymology.

This species is named after MV Tara, which allowed the exploration of coral reefs in Gambier. Moreover, the name “tara” in the Polynesian language may refer to a spiny, pointed object, which applies well to the new species typically featuring pointed skeletal structures. In the same language, Tara is also the name of a sea goddess.

## Discussion

The study of the *Echinophyllia tarae* sp. n. material and the *in situ* observations indicated a remarkable phenotypic variation within and between specimens regarding calice size, shape, and inclination, and the number of septa and paliform lobes in the corallite. Thus, *Echinophyllia tarae* sp. n. is distinct from the other *Echinophyllia* species by the presence of a larger central corallite with a raised wall, thicker primary costosepta, and a very pronounced crown of paliform lobes. In addition, *Echinophyllia tarae* sp. n. forms relatively small colonies with few corallites.

The type species of *Echinophyllia*, *Echinophyllia aspera*, has overall smaller and more evenly sized corallites than *Echinophyllia tarae* sp. n. Although a central corallite can be recognized in small colonies of *Echinophyllia aspera* (Veron and Pichon 1980), this is smaller than in the new species and does not have its very thickened septa and pronounced crown of paliform lobes. Although the few secondary corallites in *Echinophyllia tarae* sp. n. may be smaller than the central one and comparable in size to those in *Echinophyllia aspera* (Figures 5b–c, e), in the latter species the septa are equally thin, while in the former the first cycle septa are markedly thicker. Moreover, *Echinophyllia aspera* can form much larger colonies than *Echinophyllia tarae* sp. n. (see Veron and Pichon 1980, figures 516, 520). *Echinophyllia rugosa* Chevalier, 1975, from New Caledonia, has smaller corallites than *Echinophyllia aspera* and less numerous septa (Chevalier 1975). *Echinophyllia rugosa* was synonymized with *Echinophyllia aspera* by Veron and Pichon (1980), and has smaller corallites than *Echinophyllia tarae* sp. n.

Among the remainder of the *Echinophyllia* species, *Echinophyllia echinoporoides* and *Echinophyllia costata*,have smaller and more numerous corallites with less prominent costosepta ornamentation and a more poorly developed crown of paliform lobes, whereas a central larger corallite is not distinguishable (Veron and Pichon 1980, Veron 2000, 2002). Although a central corallite may be present in *Echinophyllia pectinata* and in some colonies of *Echinophyllia patula* (Veron 2000), inthese species corallites are also smaller than in *Echinophyllia tarae* sp. n. Moreover, in *Echinophyllia pectinata*, costae are equal and typically smooth (Veron 2002), corallites of *Echinophyllia patula* are typically flush with the colony surface (Hodgson and Ross 1982), and a crown of paliform lobes is absent in both species.

*Echinophyllia orpheensis* has larger corallites than any of the aforementioned *Echinophyllia* species. A larger central corallite can be observed in some specimens like in one of the paratypes (Veron and Pichon 1980: figure 525), and it forms a well-developed crown of paliform lobes and exert septa. However, when compared to *Echinophyllia tarae* sp. n., the corallites of *Echinophyllia orpheensis* are still smaller and more uniform in average diameter. Furthermore, in *Echinophyllia orpheensis*, corallites are often raised and more exsert than in the new species. They also point more irregularly in various directions and have fewer septa with smaller dentations than in *Echinophyllia tarae* sp. n.

**Figure 9.**
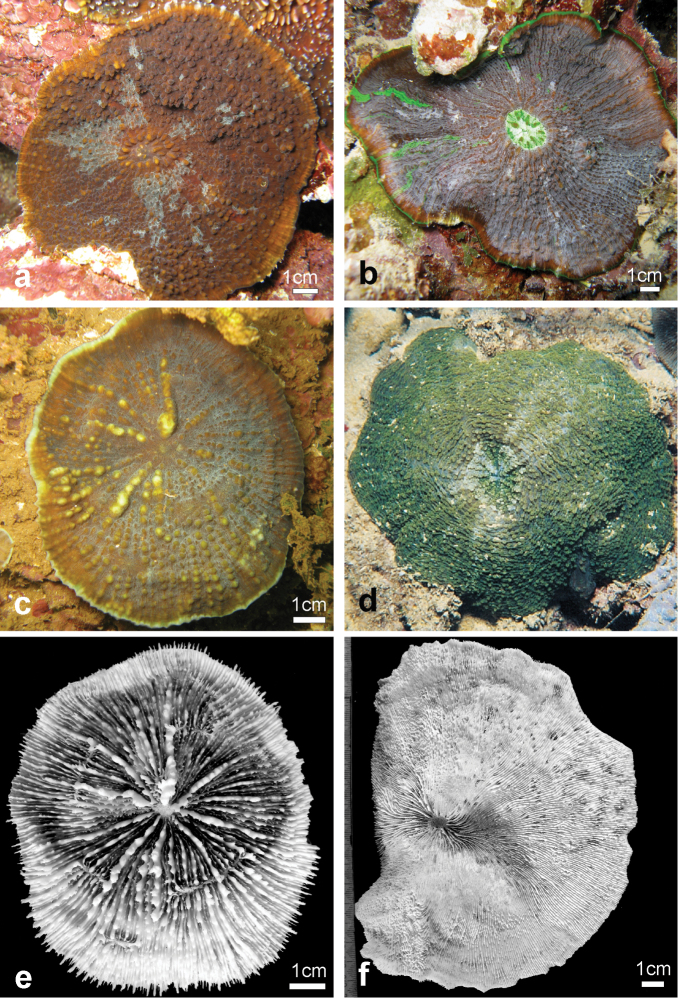
Comparison of *in situ* morphology between specimens of *Echinophyllia tarae* sp. n., *Echinophyllia echinata*, and *Echinomorpha nishihirai* and corallum morphology of the latter two **a** *Echinophyllia tarae* sp. n. resembling *Echinophyllia echinata*, Agakauitai Island (Site 10) **b** another colony of the new species resembling *Echinomorpha nishihirai*, Taravai Island (Site 11) **c** *Echinophyllia echinata* from Cap Bocage, New Caledonia (IRD HS 3171) **d** *Echinomorpha nishihirai*, Ryukyu Islands, Japan, picture by K. Yanagiya **e** same specimen as in **c**, **f** holotype of *Echinomorpha nishihirai* (MTQ G 32483), Okinawa Island, Japan. Images **c** and **e** from the IRD LagPlon database (http://lagplon.ird.nc/consultv2_5/rechSimple.faces). Sites are indicated in Figure 1.

**Figure 10.**
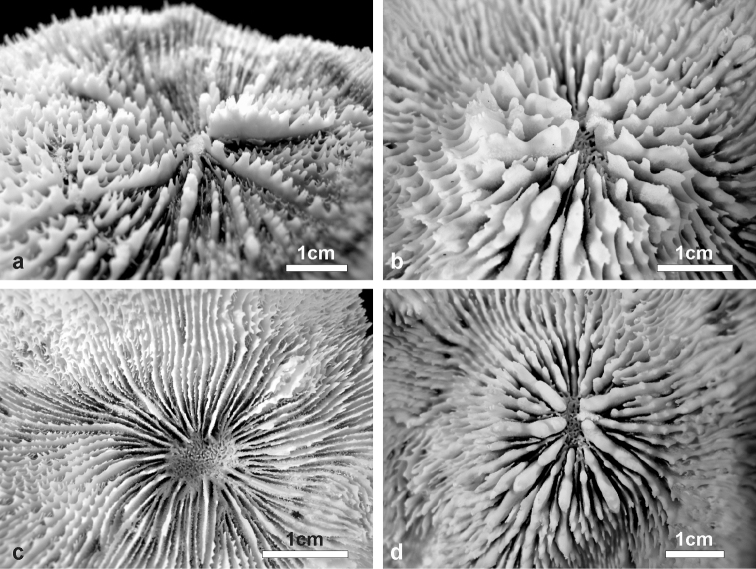
Comparison ofcentralcorallitemorphology between **a** *Echinophyllia echinata* **b** and **d** *Echinophyllia tarae* sp. n., and **c** *Echinomorpha nishihirai*: **a** side view of the central corallite of thesame *Echinophyllia echinata* specimen as in Figure 8c (IRD HS 3171) **b** side view of the central protocorallite of *Echinophyllia tarae* sp. n. (UNIMIB TO-GA099), **c** top view of the central corallite of *Echinomorpha nishihirai* holotype (MTQ G 32483) **d** top view of the same corallite as in **b**.

Live specimens of *Echinophyllia tarae* sp. n. can bear strong resemblance with *Echinophyllia echinata* (confront Figure 9a and Figure 9c) and *Echinomorpha nishihirai* (confront Figure 9b and Figure 9d). However, the skeletal morphology (Figures 9e-f, 10) helps to distinguish the new species from these two. *Echinophyllia echinata* forms thin flat to vase shaped colonies, with a conspicuous central corallite and widely spaced radials (Veron and Pichon 1980). The holotype of this species (Saville-Kent 1871) was most likely a juvenile, as also remarked by Veron and Pichon (1980). The original illustration shows the obvious larger central corallite and the thick costae continuing until the corallum margin, like in specimen IRD HS 3171 from New Caledonia (Figures 9c, e). Specimens of *Echinophyllia echinata* illustrated by Veron and Pichon (1980) were examined at the MTQ, but none of these bears close morphologic similarity with *Echinophyllia tarae* sp. n. In fact, the corallum growth form in *Echinophyllia echinata* and the presence of a larger central corallite may indeed remind of *Echinophyllia tarae* sp. n. However, the usual pattern of corallite arrangement around the central one in *Echinophyllia echinata* typically deriving from a circumoral budding (Figure 9e) is not observed in *Echinophyllia tarae* sp. n., in which peripheral budding is observed (Figures 6b, d). Furthermore, the central corallite in *Echinophyllia echinata* (Figure 10a) does not have the pronouncedly raised wall typical of *Echinophyllia tarae* sp. n. (Figure 10b). Septa in *Echinophyllia echinata* are thinner, septal teeth are smaller and more regularly spaced, and are devoid of the typical crown of well developed paliform lobes of *Echinophyllia tarae* sp. n.

*Echinomorpha nishihirai* initially described by Veron (1990) in *Echinophyllia* and later moved to a new monotypic genus, is similar to *Echinophyllia tarae* sp. n. in having a prominent central corallite, widely spaced peripheral corallites of smaller size, and by forming small encrusting colonies with a few corallites. Some of the colonies of *Echinophyllia tarae* sp. n. observed *in situ* bear resemblance with *in vivo* images of *Echinomorpha nishihirai* (Figures 9b, d). Despite this similarity, the skeletons of the two taxa are remarkably different. The holotype of *Echinomorpha nishihirai* (MTQ G 32483) was examined (Figures 9f, 10c). Although the central corallite of the largest collected specimen of *Echinophyllia tarae* sp. n. collected is similar in size to that of *Echinomorpha nishihirai*, the latter lacks the typical raised rim of the former and its septa are more numerous, thinner and with finer dentations (Figure 10c). In another specimen of *Echinomorpha nishihirai* (MTQ G 70283) the central corallite is actually more protruding from the colony surface than in the holotype, with which it shares thinner and more numerous septa reaching the columella and a denser and more acute ornamentation. Furthermore, the typical and obvious crown of paliform lobes of *Echinophyllia tarae* sp. n. is not present in *Echinomorpha nishihirai*.

In his report on the diversity and distribution of scleractinian corals of the Gambier Islands Chevalier (1974) indicated the presence of *Echinophyllia aspera* typically found on fringing reefs. Unfortunately, his publication did not include illustrations of the coral species listed by him. The author was a remarkably thorough scientists and his unpublished field notes (*cahiers de terrain*) are an example of a naturalist’s dedication and passion. He wrote these down in a series of notebooks in which he numbered pages and he registered painstakingly all details of reef profile, species distribution, specimens identification, colour and more. The complete series of notebooks is deposited at the MNHN in Paris and I could examine them during a visit in 2012. Field notes of 1969 (from page 1637 to 1707) include notes of Chevalier’s fieldwork in the Gambier and served as reference for his 1974 publication. The author collected several specimens of *Echinophyllia* sp. at different sites in the lagoon which he later identified as *Echinophyllia aspera* (Chevalier, 1974). At Agakauitai Island, he sampled specimen GAM78b which he identifies as “Echinophyllia *encroutant mais peut être espèce differente, couleur vert foncé*” [encrusting *Echinophyllia* but possibly a new species, colour dark green]. Unfortunately, despite much effort, the Gambier collection of Chevalier could not be located in the Scleractinia collection of the MNHN in Paris. Hence I was unable to verify if the possibly new species found by Chevalier is indeed the same as the one presently described, or if any of the specimens he collected belong to *Echinophyllia tarae* sp. n.

In their compilation of zooxanthellate scleractinian coral species at 19 localities in the Eastern, South-eastern, and Central Pacific Ocean Glynn et al. (2007) reported *Echinophyllia aspera* from the Society, Tuamotu, and Austral Islands in French Polynesia and *Echinophyllia echinata* from the Austral mostly based on data from Chevalier (1982) and later additions reworked by Pichon (1985). Again, in absence of a reference collection it is impossible to verify if *Echinophyllia tarae* sp. n. has been misidentified with these species and, hence, if its distribution is actually wider than presently reported.

## Conclusion

*Echinophyllia tarae* sp. n. is described from the Gambier Islands, French Polynesia. The species is characterized by a high intraspecific variation of several morphological traits. It also shows typical features that distinguish it from the other *Echinophyllia* species and from *Echinomorpha nishihirai*, such as the dimensions and the protrusion of the largest corallite (centrally located in flat colonies), the thickness of the septa, and the development of the crown of paliform lobes. Although the new species is common in the Gambier Islands, its occurrence elsewhere is unknown. The sampling of coral tissue from the type specimens of *Echinophyllia tarae* sp. n. will allow molecular analyses in order to examine its phylogenetic relationships with its congeners and other species in the Lobophylliidae.

## Supplementary Material

XML Treatment for Echinophyllia tarae
